# Nutritional strategies in abdominal surgery: the potential of ketogenic diet

**DOI:** 10.3389/fnut.2025.1716022

**Published:** 2026-01-15

**Authors:** Longjie Xu, Xiaohua Li, Chun Cao

**Affiliations:** 1 Department of General Surgery, The Second Affiliated Hospital of Soochow University, Suzhou, China; 2 Department of Thyroid and Breast Surgery, Suzhou Wuzhong People's Hospital, Suzhou, China

**Keywords:** abdominal surgery, ketogenic diet, metabolic mechanisms, perioperative period, protection mechanism

## Abstract

Metabolic stress responses in the perioperative period of abdominal surgery can lead to systemic inflammatory response syndrome and accelerate the breakdown of glycogen, lipids and proteins, which affect skeletal muscle content and have a negative impact on patient functional recovery. Comprehensive perioperative nutritional support can improve patients' preoperative physical state and reduce postoperative complications. The ketogenic diet (KD), which consists of low carbohydrate, moderate protein and high fat contents, is widely used for the treatment of obesity and neurodegenerative diseases, and recent studies have focused on the associations between KD and perioperative nutritional support for abdominal surgery. In this review, the concept of KD and its metabolic mechanisms, as well as the potential benefits of KD during the perioperative period of abdominal surgery, are discussed. In addition, the risks and challenges of KD and the corresponding solutions are presented as well. A comprehensive understanding of the mechanisms by which KD acts may provide new nutritional strategies to improve the prognosis of abdominal surgery patients.

## Introduction

Over the past few decades, abdominal surgery has undergone a significant transformation, shifting from traditional open invasive procedures to advanced closed minimally invasive techniques, which have substantially minimized surgical trauma and reduced postoperative complications (1). However, the chronic inflammation induced by underlying diseases and the surgery itself can contribute to metabolic stress responses and induce the release of inflammatory mediators (2–4). Inflammatory mediators trigger systemic inflammatory response syndrome, which leads to the catabolism of glycogen, lipids and proteins, thereby diverting protein from maintaining peripheral muscle mass to promote healing and enhance immunity (5, 6). The loss of skeletal muscle has a negative impact on functional recovery in patients after abdominal surgery (7, 8). Inflammatory mediators have also been shown to regulate skeletal muscle metabolism through two mechanisms, the first by directly influencing protein synthesis and degradation and the second by indirectly inhibiting the activity of regulatory anabolic hormones and activating the hypothalamic–pituitary–adrenal axis (9, 10). Moreover, surgical stress increases blood levels of cortisol, which provides amino acid precursors for gluconeogenesis, wound healing and immune functions through promoting the breakdown of proteins into amino acids (11, 12). Perioperative malnutrition is closely associated with increased postoperative complication rates, prolonged hospital stays and reduced long-term survival in abdominal surgery patients (13, 14). Given that malnutrition is an important risk factor for postoperative complications, nutritional support can reduce the incidence of surgical site infections (SSIs), organ dysfunction and other complications, especially among malnourished patients or those undergoing high-risk surgeries and European Society for Clinical Nutrition and Metabolism also recommends that nutritional support should be provided at the onset of malnutrition in patients undergoing abdominal surgery (15–17).

The ketogenic diet (KD), which consists of low carbohydrate, moderate protein and high fat contents, is widely used for the treatment of obesity and neurodegenerative diseases (18–22). Patients receiving a very low-calorie KD for 12 months can achieve greater weight loss than those receiving a low-fat diet (19). A prospective clinical study including 30 patients with morbid obesity indicates that the administration of a 2-week KD before abdominal surgery contributes to significant weight loss compared with the administration of a Mediterranean diet (23). Moreover, patients receiving KD after pancreatectomy for pancreaticobiliary cancer experience significant improvements in the levels of cancer-related metabolites (24). KD limits the level of glucose while increasing the level of ketone bodies in serum, which not only limits the progression of cancer but also provides normal tissues with adequate ketone bodies, thereby exerting anticancer effects (25). KD significantly differs from standard nutritional strategies used in enhanced recovery after surgery (ERAS) protocols due to its unique macronutrient composition, physiological effects and metabolic outcomes. KD forces the body into a state of ketosis, where ketone bodies become the primary energy substrate instead of glucose. While conventional ERAS protocols emphasize carbohydrate loading preoperatively which replenishes glycogen stores, minimizes catabolism, and reduces perioperative insulin resistance (26). By drastically reducing carbohydrate intake, KD minimizes blood glucose fluctuations and suppresses insulin secretion. This metabolic shift reduces insulin resistance and improves glycemic control, potentially beneficial for patients with metabolic dysfunctions such as type 2 diabetes and obesity (27). Nonetheless, despite being a potential therapy for abdominal surgery (28, 29), there is presently a lack of a narrative review that evaluates the effects of KD during the perioperative period of abdominal surgery. In this review, we first demonstrate the concept of KD and its metabolic mechanisms. Second, we discuss the potential benefits of KD during the perioperative period of abdominal surgery. Finally, we present the risks and challenges of KD and the corresponding solutions.

## Methods

For this review, a systematic search is carried out for all relevant articles in the PubMed and Scopus database without imposing any time restrictions. The literature search covers the following keywords: (“ketogenic diet” OR “low carbohydrate ketogenic diet” OR “ketone bodies”) AND (“abdominal surgery” OR “Glycometabolism” OR “protein metabolism” OR “lipid metabolism” OR “bariatric surgery” OR “malnutrition” OR “liver dysfunction” OR “ischemia–reperfusion injury” OR “the gut microbiota” OR “abdominal tumors”). Inclusion criteria: (1) Studies focusing on the application of KD in the perioperative period of abdominal surgery, including preoperative preparation, intraoperative support, and postoperative recovery; (2) Studies involving metabolic mechanisms, efficacy, safety, and clinical outcomes related to KD; (3) Original Studies, including clinical trials, animal experiments and *in vitro* studies. Exclusion criteria: (1) Studies unrelated to abdominal surgery or perioperative periods; (2) Case reports, letters to the editor, or abstracts without full-text data. Subsequently, the relevant articles are subject to classification, critical evaluation and summarization. Since the current studies on KD in abdominal surgery involve diverse study types (clinical, animal, and *in vitro*) and heterogeneous outcomes (metabolic indicators, complication rates, and organ function), making it difficult to conduct the meta-analysis. A narrative review can more comprehensively integrate multi-type evidence and systematically sort out mechanisms and clinical applications. Limitations of the search strategy: (1) Only PubMed and Scopus databases are searched, potentially missing relevant studies from other databases; (2) Non-English language studies are not included, which may have led to information bias.

### The conception of KD

The KD is a high-fat, moderate-protein, low-carbohydrate dietary pattern, which could induce ketosis, thereby improving the nutritional state (30). The classic KD suggests that the ratio of grams of fat to total grams of carbohydrates and proteins is typically 4:1 or 3:1, whereas the ratio is 2:1 or 1:1 in the modified KD (31).

### Metabolic mechanisms of KD

In a normal diet, the body relies primarily on carbohydrates for energy (32). When a KD is adopted, carbohydrate intake is extremely low, and the body is forced to switch from a glucose-based mode of energy supply to one in which ketone bodies, including acetoacetate (AcAc), beta-hydroxybutyrate (BHB) and acetone, produced by fat metabolism are the main energy source (33, 34). Fatty acids in the liver are oxidized via β-oxidation to form acetyl coenzyme A (acetyl-CoA) (35). Owing to low carbohydrate intake and insufficient oxaloacetate, acetyl-CoA cannot enter the tricarboxylic acid (TCA) cycle for complete oxidation, thus condensing to form ketone bodies (36, 37). Ketone bodies can cross the blood–brain barrier to supply energy when glucose is in short supply (38, 39). Moreover, BHB exerts anti-inflammatory effects by reducing the expression of lipopolysaccharide-induced pro-inflammatory proteins via the nuclear factor kappa-B (*NF-*κ*B*) pathway and induces intestinal cell differentiation via the mammalian target of rapamycin (*mTOR*) pathway (40, 41). However, AcAc in diabetic patients has a pro-inflammatory effect through inducing the expression of tumor necrosis factor-α (TNF-α), monocyte chemotactic protein-1 (*MCP-1*) and the production of reactive oxygen species (ROS) (42, 43).

### Glycometabolism

During the perioperative period, patients often experience stress hyperglycemia (44). The KD reduces blood glucose fluctuation due to low carbohydrate intake (45). A clinical study reveals that patients who undergo abdominal surgery have significantly lower glycated hemoglobin levels with the administration of KD (46). A randomized, single-blinded, placebo-controlled, crossover study indicates that BHB, the metabolite of KD, abrogates insulin resistance through binding to hydroxy-carboxylic acid 2 receptors and inhibiting lipolysis (47). Preclinical studies indicate that mice fed with KD for 4 weeks present increased absolute numbers of *Bifidobacteria*, which have the potential to reduce glucose intolerance in diabetic mice (48, 49). The acute administration of BHB can increase the level of serum insulin and decrease the level of serum glucagon in both mouse and human islets, whereas the chronic administration of BHB has little effect on the secretion of insulin in human islets (50). Furthermore, BHB can also increase the level of serum insulin through potentiating hepatic insulin signaling, thereby inhibiting the production of hepatic glucose and improving glucose homeostasis (51).

### Protein metabolism

In the early stages of the administration of KD, the body undergoes a series of metabolic adaptations due to the drastic reduction in carbohydrate intake (52, 53). At this time, the body may experience a transient negative nitrogen balance (54). This is due to the altered metabolic state of some tissues and organs as the shift from a glucose-based metabolic pattern to ketone bodies as the main source of energy (33). The deficiency of amino acid has a negative impact on protein synthesis in the early stages of the administration of KD (55, 56). As the KD continues, the body gradually adapts to the new pattern of energy metabolism, and the nitrogen balance changes (57, 58). At this time, the body uses the ingested protein more efficiently and reduces unnecessary protein catabolism. The synthesis and breakdown of muscle protein reach a new state of equilibrium, where the needs for amino acids for tissue repair and enzyme and hormone synthesis are met without the excessive breakdown of muscle protein (58).

### Lipid metabolism

To adapt to energy demands, KD modulates the expression and activity of lipases, which results in the breakdown of triglycerides into glycerol and fatty acids within fat cells (59–61). Following their transportation to the liver, the activity of the fatty acid oxidase system in the liver is enhanced, which breaks down fatty acids into acetyl-CoA through the β-oxidation pathway to provide energy for the body (62). Owing to inadequate carbohydrate intake, oxaloacetate production is reduced, which contributes to the inability of acetyl-CoA to undergo the TCA cycle, thereby participating in the synthesis of ketone bodies in the liver (36). In addition, KD can reduce the accumulation of visceral fat, which is strongly associated with the development of metabolic diseases (63). Several studies have indicated that people receiving KD over a long period of time have significantly lower levels of visceral fat and better metabolic state (64, 65).

### KD and bariatric surgery (BS)

BS has become the most effective treatment for severe obesity (66). The most common surgical procedures are laparoscopic Roux-en-Y gastric bypass (RYGB) and sleeve gastrectomy (67). Patients with high visceral fat and liver fat accumulation are more likely to have postoperative pulmonary infection, abdominal infection or gastrointestinal bleeding after BS surgery (68). KD can safely and efficiently reduce the weight of patients before BS, thereby improving the outcomes of patients undergoing BS (66). Compared with a standard low-calorie diet, the administration of very low-calorie KD before BS for 2 months can reduce visceral and liver fat accumulation (65). A prospective pilot study demonstrates that 27 morbidly obese patients have significant decreases in body weight and left hepatic lobe volume and improved micronutrient state after a 4-week preoperative ketogenic micronutrient-enriched diet, which can improve the physical state of patients before BS (69). Preoperative administration of KD can significantly decrease visceral fat, thereby reducing the risk of anastomotic leakage and incisional SSIs (65, 70). A prospective clinical study reveals that body weight, body mass index, waist circumference and neck circumference significantly decrease after 10 days of very-low-calorie KD in patients scheduled for BS, which indicates that a very low-calorie KD is safe and effective in regulating the physical state of patients before BS (71). Moreover, compared with a low-calorie diet, the administration of a very low-calorie KD for 3 weeks before BS results in better surgical outcomes, including postoperative drainage output and hemoglobin (72). A randomized multicenter comparative study reveals that patients who receive a 4-week low-calorie KD combined with continuous positive airway pressure have significantly lower C-reactive protein levels than patients who receive continuous positive airway pressure alone (73). KD also plays a role in reducing preoperative levels of glycemia and insulin in patients with obesity, which contributes to a smooth surgical procedure and less postoperative infection (74–76). The administration of a very low-calorie KD for 8 weeks can also significantly improve the gut microbiota and metabolic parameters in patients undergoing BS (77). KD offers a more efficient and safer pathway for surgical treatment in obese patients by reducing fat accumulation, stabilizing metabolic parameters and alleviating postoperative inflammation, but standardized KD protocols, including calorie intake and duration, are lacking. Future trials should define optimal KD regimens for BS.

### KD and malnutrition after abdominal surgery

Patients who have undergone abdominal surgery are likely to experience elevated levels of oxidative stress and increased energy expenditure (78). KD is characterized by a high fat content, which results in the production of higher levels of energy than carbohydrates and proteins (30). Therefore, KD has the capacity to provide adequate calories to support metabolism and recovery in patients after abdominal surgery. In addition, KD increases the level of ketone bodies in the serum, which can provide the brain and other important organs with a consistent source of energy (30). A retrospective study demonstrates that the administration of a very low-calorie KD before abdominal surgery reduces drainage outputs and increases the level of hemoglobin through the provision of adequate nutrition (72). The body needs more protein to repair damaged tissue and promote wound healing after surgery. A prolonged postoperative KD has been shown to reduce the amount of protein consumed for energy and increase the availability of protein for muscle repair and recovery by utilizing fat as a source of energy (57). The obese patients who receive a very low-calorie KD for 12 months experience significant weight loss without significant loss of muscle mass (79). Preclinical studies indicate that KD plays a role in regulating the levels of hormones, particularly insulin and growth hormone (47, 80). Both insulin and growth hormone induce protein synthesis via the activation of insulin-like growth factor 1 (*IGF-1*) signaling (81). KD promotes protein synthesis by alleviating insulin resistance and increasing the amount of glucose transporter type 4 on muscle cell membranes (80). KD can also increase the level of growth hormone by regulating the level of plasma ghrelin, thereby stimulating the growth of muscle (82). In addition, KD can change the structure of the gut microbiota and promote the growth of beneficial bacteria, which can promote the absorption of nutrients through improving intestinal barrier function and enhancing intestinal innate immunity (83).

The diet containing a high proportion of fat can promote the absorption of fat-soluble vitamins, such as vitamins A, D, E and K (84). Over than half of the patients who undergo BS suffer from vitamin D deficiency within 10 years after surgery, which usually results in delayed wound healing and infection. KD can promote the absorption of vitamin D to prevent these complications by regulating re-epithelialization and innate immunity (85). Preclinical studies indicate that consecutive gavage of vitamin A for 7 days can reverse dexamethasone-induced tracheal anastomosis injury in rats (86). Vitamin E, such as γ-tocopherol, δ-tocopherol and γ-tocotrienol, inhibits proinflammatory signals such as *NF-*κ*B* and signal transducer and activator of transcription (*STAT*) −3/6 by scavenging reactive nitrogen and inhibiting cyclooxygenase- and 5-lipoxygenase-catalyzed eicosanoids, which is beneficial for postoperative recovery (87). Vitamin K administered by subcutaneous injection significantly improves colonic wound healing in rats undergoing colostomy (88). KD provides a comprehensive metabolic support framework for preoperative energy supply and postoperative recovery by promoting adequate utilization of fats and proteins, optimizing gut health and enhancing absorption of fat-soluble vitamins.

### KD and liver dysfunction

Postoperative liver dysfunction is a potential complication following abdominal surgery (89). A study including 10 participants reveals that the administration of KD for 6 days significantly decreases intrahepatic triglyceride, γ-glutamyl transpeptidase and alkaline phosphatase levels (90). In addition, patients with obesity achieve significant improvement in liver steatosis when they receive a very low-calorie KD for 2 months compared with those receiving a low-calorie diet (65). Moreover, KD decreases the levels of aspartate aminotransferase and alanine aminotransferase in patients with liver dysfunction after diet intervention for 12 weeks (91).

Hepatic encephalopathy (HE) is defined as brain dysfunction caused by liver dysfunction and/or portosystemic shunts and is a serious complication after liver surgery (92, 93). In patients with HE, the ability to metabolize carbohydrates is reduced, which leads to a lack of energy supply to the brain (94). While KD provides alternative energy for the brain by increasing the concentration of ketone bodies which improve the energy supply of the brain (34). KD also decreases the incidence of HE through directly reducing the synthesis of ammonia and indirectly inhibiting the growth and reproduction of ammonia-producing bacteria (32, 95, 96). Furthermore, BHB can improve cognitive performance in patients with HE by decreasing the level of gamma-aminobutyric acid in the brain (97, 98). KD provides significant support for perioperative patients by improving liver function, reducing hepatic fat accumulation and regulating cerebral energy supply, which demonstrates particular efficacy in the prevention of postoperative HE.

### KD and ischemia–reperfusion (I/R) injury

I/R injury is defined that tissues and organs restore blood perfusion after a period of ischemia, which not only fails to restore the functions of tissues and organs but also exacerbates metabolic disorders and structural damage. Abdominal surgery, especially organ transplantation, has a high risk to cause I/R injury (99–102). The potential mechanisms of I/R injury include the following: (1) Mitochondrial respiratory dysfunction and a reduction in the activity of endogenous antioxidants during hypoxia lead to the production of large amounts of ROS (103); (2) Intracellular calcium overload aggravates mitochondrial dysfunction and inhibits the production of adenosine triphosphate (104, 105); (3) I/R activates inflammasome and releases large amounts of inflammatory cytokines, which can further activate the inflammatory cascade response. In kidney transplant patients, KD can increase the glomerular filtration rate, delay the progression of chronic kidney disease, and no adverse reactions such as hypercalcemia or protein malnutrition have been observed (106). Preclinical studies indicate that the activation of the mitochondrial permeability transition pore (*mPTP*) results in mitochondrial dysfunction, swelling and rupture of the outer membrane with subsequent release of cytochrome c, which aggravates liver I/R injury (107–111). KD can inhibit *mPTP* opening by imitating the conventional *mPTP* inhibitor cyclosporin A and prevent mitochondrial *mPTP* activation by increasing the threshold for calcium-induced *mPTP* (112, 113). Meanwhile, KD enhances cellular resistance to oxidative stress through inducing the expression of intracellular antioxidant enzymes, such as superoxide dismutase and glutathione peroxidase (39). Moreover, KD can reduce the production of ROS through inhibiting the expression of nicotinamide adenine dinucleotide phosphate oxidase during I/R injury (114–116). In the inflammatory cascade triggered by I/R injury, proinflammatory cytokines, such as *TNF-*α*, interleukin (IL)-1*β and *IL-6*, are released in large quantities (102). KD downregulates the levels of these proinflammatory cytokines, thereby reducing the intensity of the inflammatory response (117). For example, in the renal I/R injury, the expression of *TNF-*α*, IL-6* and *MCP-1* in the kidney cortex is significantly reduced with the administration of KD (115). KD attenuates renal I/R injury in mice by reducing oxidative stress and inflammation and that BHB attenuates renal I/R injury in mice through its antipyroptotic properties (115, 118). I/R injury promotes the transcription of multiple inflammatory genes through activating *NF-*κ*B*, a key regulator of the inflammatory response (119, 120). KD blocks the transmission of inflammatory signals by inhibiting the activation of *NF-*κ*B*, thus reducing the degree of tissue damage caused by the inflammatory response (121, 122). KD exerts multifaceted mechanisms in mitigating I/R injury by suppressing inflammatory responses, reducing oxidative stress and safeguarding mitochondrial function, but clinical trials are needed to validate these benefits in organ transplantation or liver resection patients.

### KD and the gut microbiota

Abdominal surgery can significantly alter the gut microbiota, leading to numerous complications, including SSIs, abdominal abscesses and heightened inflammatory responses (123–125). SSIs are a common complication following abdominal surgery and usually caused by *gram-negative bacilli* and *facultative anaerobes* (126, 127). A recent study indicates that KD could reduce the abundance of these bacteria, thereby potentially lowering the risk of SSIs (72). A reduced abundance of *Bifidobacterium* in patients with RYGB can impair immune function, anti-inflammatory capacity, glycemic control and insulin sensitivity, but KD has been shown to effectively restore *Bifidobacterium* levels, potentially improving postoperative outcomes (49, 128–131). Moreover, KD triggers an increase in the abundance of *Akkermansia*, thereby contributing to the improvement of intestinal barrier function and the enhancement of intestinal immunity (132, 133). KD regulates the community structure of bile salt hydrolase-active bacteria, thereby controlling microbial bile acid metabolism and reducing caloric absorption, which helps prevent obesity (134, 135). Preclinical studies indicate that mice receiving KD for 4 days present a greater abundance of *Parabacteroides*, which plays a role in improving anastomotic healing by inhibiting the production of inflammatory factors, such as macrophage inflammatory protein-1α and −2, *IL-17A/F* and *MCP-1* (136). KD regulates the gut microbiota, altering the production patterns of short-chain fatty acids (SCFAs) like butyrate, propionate and acetate (137, 138). SCFAs play distinct roles in maintaining intestinal function: butyrate serves as a key energy source for intestinal epithelial cells and regulates intestinal immunity and inflammation (139), while propionate promotes the differentiation of intestinal crypt cells and supports intestinal homeostasis (140). Intestinal I/R injury-induced gut microbiota dysbiosis, marked by *Escherichia coli* overgrowth and *Lactobacillus* decline, can be mitigated by KD, which leads to a restore in intestinal function (83, 123, 141).

However, the association between KD and the gut microbiota is under debate. A recent study demonstrates that KD could reduce the abundance of beneficial gut bacteria, such as *Lactobacillus, Lactococcus* and *Coprococcus*, and increase the abundance of pathogenic bacteria, such as *Anaerofilum, Enterococcus, Roseburia* and *Enterobacter* (142). KD's effects on gut microbiota are inconsistent, possibly due to variations in KD protocols and patient populations. Future studies should clarify microbiota changes in specific abdominal surgery subgroups to develop personalized KD strategies.

### KD and abdominal tumors

Recently, an increasing number of studies have focused on KD as an adjuvant therapy for abdominal tumors. The majority of abdominal tumors predominantly depend on glucose as their primary energy substrate and lack the ability to metabolize ketone bodies. In contrast, normal tissue cells exhibit metabolic flexibility and are capable of switching to ketone bodies as alternative energy sources when glucose is limited (143). Although KD does not directly exert anticancer effects, it has the potential to increase the sensitivity of tumor cells to cytotoxic chemotherapy. There are two potential mechanisms explaining this effect: first, both the KD and conventional chemotherapy agents increase the NADH/NAD ratio in tumors through the modulation of redox reactions, resulting in compromised tumor oxygen supply or mitochondrial dysfunction; second, KD triggers an inflammatory response by activating the interferon pathway and the *IL-6/Janus Kinase/STAT-3* signaling pathway, ultimately promoting tumor fibrosis (144). Sphingolipids, which are pancreatic cancer-associated metabolites, are reportedly involved in tumor signaling networks and play a role in regulating tumor growth, proliferation and migration (145). A pilot study reveals that the administration of a low-calorie KD for 2 weeks in patients undergoing pancreatectomy for pancreaticobiliary cancer significantly reduces sphingolipid levels compared with those in the control group (24). The combination of KD with hyperthermia and hyperbaric oxygen therapy has been shown to markedly suppress the progression of advanced gastric cancer (146). Preclinical studies indicate that KD in combination with gemcitabine maintains muscle strength and alleviates pancreatic cancer-associated cachexia by inhibiting autophagy and increasing the level of eukaryotic translation initiation factor 2α phosphorylation in mice (147). The aberrant activation of the phosphatidylinositol 3-kinase/protein kinase B/*mTOR* pathway contributes to the regulation of tumorigenesis, cancer metabolism and chemotherapy resistance (148). KD combined with gemcitabine can inhibit this pathway, thus exerting an antitumor effect (149). Moreover, the development of cachexia in advanced tumors is correlated with *IGF-1*, a critical factor that negatively regulates protein synthesis (150). KD reduces the serum level of *IGF-1*, to alleviate tumor-induced cachexia (151). Moreover, KD plays a role in promoting ketosis, which inhibits hyperglycemia-induced *mTOR* upregulation, thereby suppressing the proliferation and growth of tumors (152). Compared with those subjected to cytotoxic chemotherapy alone, mice administered KD alongside cytotoxic chemotherapy present a significant reduction in tumor volume (144). Additionally, compared with control mice, mice receiving KD and gemcitabine, particularly female mice, have longer overall survival (149). Furthermore, pancreatic cancer-bearing mice fed KD in combination with gemcitabine exhibit little hepatotoxicity, indicating the protective effect of KD on the liver (153). The administration of a 12-week KD in rats improves the efficacy of chemotherapy in treating gastric tumors through enhancing autophagy, oxidative stress, and miR-340-mediated apoptosis (154). KD also shows promising potential in the treatment of liver tumors. A recent study shows that KD inhibits the proliferation and migration of liver tumors by reducing the production of insulin and downregulating the expression of forkhead box C2 (155). In addition, KD can increase the expression of hydroxymethylglutaryl-CoA synthase 2, contributing to the suppression of liver tumors (156). KD induces programmed death-1 overexpression on the surface of renal tumor cells, thereby exerting its potential to inhibit the growth and proliferation of renal cancer (157). As an adjunctive therapy, KD holds considerable promise in perioperative management of abdominal tumors through regulating metabolic pathways, alleviating cachexia, protecting normal tissues and enhancing chemotherapy sensitivity, but large-scale clinical trials are needed to validate its efficacy in different tumor types and optimize combination strategies with chemotherapy.

### The risk and challenge of KD during the perioperative period of abdominal surgery

KD faces multifaceted translational challenges in perioperative abdominal surgery. KD typically demands strict carbohydrate restriction, which may conflict with patients' dietary habits and cultural backgrounds, leading to poor adherence. Long-term compliance is vital for achieving and sustaining ketosis. For patients undergoing abdominal surgery, preoperative and postoperative experiences such as loss of appetite, nausea, or gastrointestinal discomfort further complicate adherence to a rigorous KD. In addition, during the initial phase of KD, depletion of glycogen stores and reduced level of insulin decrease renal sodium reabsorption, potentially leading to water and electrolyte losses, particularly sodium, potassium, and magnesium (158). Meanwhile, the prolonged administration of KD alone limits the intake of vitamins and minerals, potentially leading to nutrient deficiencies after abdominal surgery (32). During the perioperative period of abdominal surgery, patients experience an increased metabolic state, which elevates their nutritional requirements. Insufficient or imbalanced nutrient intake during this critical period can impair wound healing and hinder overall recovery (159). Initiating KD early may help patients achieve ketosis preoperatively, potentially improving metabolic status and reducing the incidence of postoperative hyperglycemia (160). However, it is crucial to take into account clinicians' professional training in nutrition to improve patients' compliance with KD (161). It may be more challenging to initiate KD immediately postoperatively, as patients may be stressed and have reduced tolerance for oral intake. Consequently, selecting appropriate timing of preoperative and postoperative implementation to maximize the benefits of KD while minimizing potential risks remains a critical issue in clinical practice. In addition, KD, characterized by extremely low carbohydrate content, results in minimal dietary fiber, which usually contributes to irregular peristalsis and impaired intestinal tract function (162, 163). Therefore, patients with long-term KD may experience intestinal dysfunction, such as constipation, bloating and abdominal pain (162). The higher level of fat in KD may lead to excessive production of ketone bodies, triggering ketoacidosis, which is a serious metabolic disorder and has a negative effect on the outcomes of patients undergoing abdominal surgery (164). Moreover, KD can lead to decreased uric acid excretion, potentially causing hyperuricemia, which is associated with an elevated risk of renal impairment and cardiovascular complications (165).

To implement perioperative KD safely and effectively, standardized monitoring parameters must be established to assess ketosis status, metabolic response and potential adverse effects. BHB, the main components of ketone bodies, directly reflects the depth and maintenance of ketosis. The level of BHB can be monitored via blood or urine testing to ensure patients remain in a state of nutritional ketosis rather than ketoacidosis. Typically, plasma BHB concentrations of 0.5–3.0 mmol/L are considered indicative of nutritional ketosis (166). Monitoring blood glucose levels is vital for assessing KD's metabolic impact. Continuous glucose monitoring provides dynamic insights into glycemic fluctuations, facilitating timely adjustments to dietary and therapeutic regimens. Nitrogen balance serves as a key indicator of the relationship between protein intake and breakdown, reflecting net protein storage or consumption within the body. Nitrogen balance monitoring enables the evaluation of whether KD provides sufficient protein to prevent muscle wasting and promote recovery.

### Prospects for KD in the perioperative period of abdominal surgery

Despite the risks associated with KD in the perioperative period of abdominal surgery, its potential benefits may be better realized with comprehensive nutritional support. Future studies could further explore the optimal formulation for the application of KD in the perioperative period of abdominal surgery. In addition, by incorporating additional dietary fiber and other essential nutrients into the KD, postoperative nutritional deficiencies can be effectively prevented.

## Conclusion

KD shows great potential in the perioperative period of abdominal surgery, with benefits in metabolic regulation, anti-inflammatory effects, organ protection, and gut microbiota modulation. It also serves as a promising adjuvant therapy for abdominal tumors. However, KD faces challenges such as nutrient deficiencies, adherence issues and electrolyte imbalance, which needs personalized protocols and close monitoring.

Future research should focus on: (1) validating preclinical mechanisms in human samples; (2) optimizing KD protocols, including calorie intake and duration, for different patient subgroups; (3) conducting large-scale randomized controlled trials to confirm clinical efficacy and endpoints, such as postoperative complications, muscle preservation, and inflammatory markers. With further refinement, KD may become an important part of perioperative nutritional support, improving the prognosis of abdominal surgery patients.
